# Microwave assisted the short time clean synthesis of 1,3-diketones as building blocks in heterocyclic synthesis: a facile synthesis and antimicrobial evaluation of new dihydropyridine, 4H-pyrane, dihydropyridazine, pyrimidine and pyrazole derivatives

**DOI:** 10.1007/s11030-020-10152-9

**Published:** 2021-01-05

**Authors:** Mohamed Ahmed Mahmoud Abdel Reheim, Ibrahim Saad Abdel Hafiz, Hend Saad Eldin Abdel Rady

**Affiliations:** Department of Chemistry, Faculty of Science, Arish University, Arish, 45511 Egypt

**Keywords:** Heterocycles, Microwave irradiation, Spectral characteristics, Antimicrobial activities

## Abstract

**Abstract:**

The compounds bearing naphthalene moiety can be used as medical preparations because of their wide spectrum of biological activity and low toxicity. In this study, a new series of azoles or azines were synthesized from the reaction of the key intermediate 1-(1-hydroxynaphthalen-2-yl)-3-phenylpropane-1,3-dione **3** with a variety of electrophilic and nucleophilic reagents under a variety of mild conditions. The chemical structures of these compounds were confirmed by various spectroscopic methods such as (IR, ^1^H-NMR, ^13^C-NMR, mass spectra and elemental analyses). The prepared compounds were screened in vitro for their anti-microbial activity against some species of Gram-positive bacteria *(Staphylococcus aureus* and *Bacillus subtilis)* and Gram-negative bacteria (*Escherichia coli* and *Pseudomonas aeuroginosa*). Anti-fungal activities of the compounds were tested against yeast and mycelial fungi,*Candida albicans* and *Aspergillus flavus*. The antimicrobial activity of this series was showed either weak or moderate activities.

**Graphic abstract:**

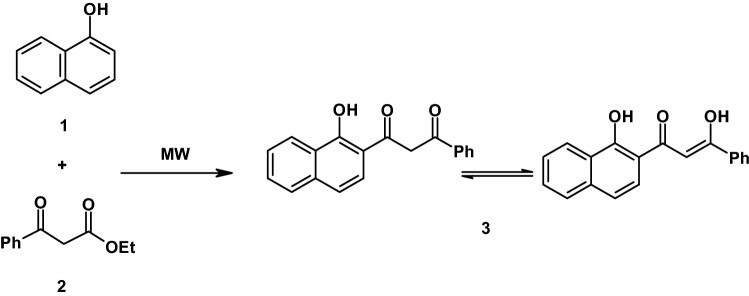

## Introduction

According to the literature survey, heterocyclic compounds containing nitrogen, sulfur and oxygen atom are found to possess a varity of biological activities. Among them, pyrazoles, cyanopyridines, pyrimidinethiones. pyranes or their fused ring systems and pyridazine are found to exhibit a wide spectrum of biological activities. Various biological applications have been reported for pyrazoles such as anti-viral [1], anti-cancer [2], anti-microbial [3], anti-inflammatory [4], anti-depressant [5] anti-convulsant [6] analgesic and antiplatelet [7, 8]. Cyanopyridine derivatives have attracted considerable attention as they appeared of interest to possess anti-cancer [9], anti-convulsant [10] and anti-microbial [11]. Pyrimidinethiones have been found to possess anti-tubercular [12]. Pyrane and fused 4H-pyrane derivatives have attracted a great interest owing to their anti-microbial [13, 14], anti-oxidant [15], inhibitors of influenza virus sialidases [16], mutagenic activity [17], anti-cancer [18] and anti-viral [19]. Also, Pyridazine ring is a nucleus of a many of drugs available in the market like cadralazine (anti-hypertensive), minaprine (anti-depressant), hydralazine (smooth muscle relaxant), pipofezine (tricyclic antidepressant) [20, 21]. The aim of the present work, was to synthesize new pyrazole, pyridine, pyrimidine, pyrane and pyridazine derivatives by using 1-(1-hydroxynaphthalen-2-yl)-3-phenylpropane-1,3-dione **3** as the key starting material. Followed by anti-microbial evaluations of newly synthesized products were done. In general, the novel synthesized compounds showed a moderate antimicrobial activity against the previously mentioned microorganisms.

## Results and discussion

**Scheme 1:**
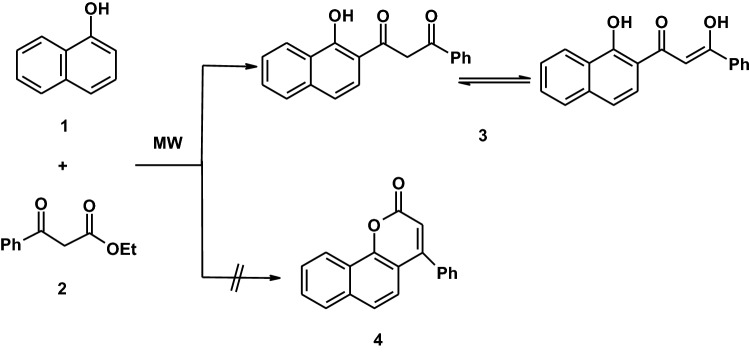
The synthesis route of compound **3**

Organic synthesis by using microwave irradiation (MW) is a new and interesting technique and is becoming popular now. The reactions under microwave irradiation take place in few minutes and no solvent is required [22–25]. In continution to our research for chemistry developments [26–29], we report here the use of microwave irradiation for the synthesis of 1-(1-hydroxynaphthalen-2-yl)-3-phenyl-propane-1,3-dione **3** in a quantitative yield (91%) from the reaction of α- naphthol and ethyl benzoylacetate in the presence of zinc chloride [30]. Rather than the expected coumarin** 4** as the literature [31]. The key intermediate **3** was prepared previously in the literature [32, 33]. The structure of the latter product **3** was established on the basis of its elemental analyses and spectral data and chemical transformation. Thus, the infrared spectrum of compound **3** revealed an absorption bands at 3365, 3061, 2924, 1720, 1639 cm^−1^ for hydroxyl, aromatic, aliphatic and carbonyl function groups, respectively. The ^1^H-NMR spectrum of compound **3** showed the following signals at *δ* = 4.49 (s, 2H, CH_2_), 7.20–8.68 (m, 11H, aromatic H), 8.69 (s, 1H, OH). Also, the mass spectrum of compound **3** is in agreement with the proposed structure, its showed a molecular ion peak at *m*/*z* = 290 (M^+^) corresponding to a molecular formula C_19_H_14_O_3_ (Scheme 1).

**Scheme 2:**
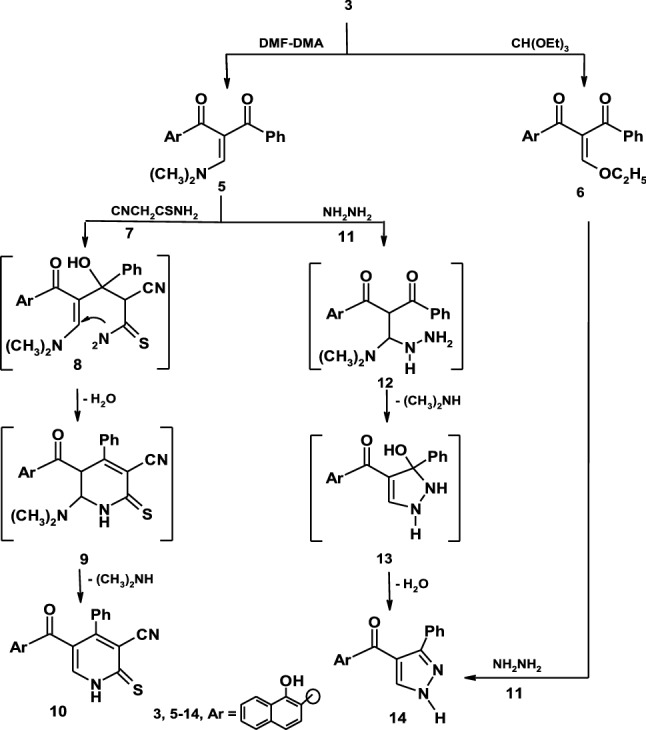
Synthetic route to pyridinethione and pyrazoles

The active methylene group in compound **3** was exploited to synthesize some novel heterocyclic compounds by its reaction with some electophilic and nucleophilic reagents. Thus, the reaction of compound **3** with dimethylformamide-dimethylacetal (DMF-DMA) in dioxane afforded the enaminone derivative **5** in a good yield as demonstrated in (Scheme 2). Establishing the structure **5** was based on its elemental analyses and spectral data. Thus, the ^1^H-NMR spectrum of compound **5** showed the following signals, a singlet signal at *δ* = 3.00 ppm assigned to N(CH_3_)_2_, a singlet signal at *δ* = 7.19 ppm assigned to oleffinic proton, a multiplet signals at *δ* = 7.64–8.67 ppm assigned to aromatic protons and a singlet signal at *δ* = 8.68 ppm assigned to hydroxyl group. Moreover, the mass spectrum revealed a molecular ion peak at *m*/*z* = 345 (M^+^) related to a molecular formula C_22_H_19_NO_3_. Also, when compound **3** was alkylated with triethylorthoformate in refluxing acetic anhydride afforded the ethoxymethylene derivative **6**. Establishing the structure **6** was based on spectral data in addition of elemental analyses. So, its ^1^H-NMR spectrum in DMSO-d_6_ revealed the following signals at *δ* = 1.11 (t, 3H, CH_3_), 4.19 (q, 2H, CH_2_), 6.33 (s, 1H, CH-oleffinic), 7.29–8.23 (m, 11H, aromatic H), 8.68 (s, 1H, OH). The mass spectrum of the same product is in accordance with the proposed structure. Thus, it showed a molecular ion peak at *m*/*z* = 346 (M^+^).

Compound **5** readily reacted with cyanothioacetamide **7** as example of active methylene reagents in refluxing ethanolic sodium ethoxide yield the expected pyridinethione derivative **10** which established on its spectral data (IR, ^1^H-NMR, mass spectra, ^13^C-NMR and elemental analyses). So, its mass spectrum revealed a molecular ion peak at *m*/*z* = 384 (M^+^ + 2) corresponding to a molecular formula C_23_H_14_N_2_O_2_S. Formation of pyridinethione **10** is believed to be proceed via initial addition of active methylene moiety in cyanothioacetamide on the π-bond system of the carbonyl group of **5** to afford the Michael adduct **8** that cyclizes by losing water molecule under the same reaction condition to give intermediate **9** which eliminate dimethylamine molecule to yield the final structure **10** as demonstrated in (Scheme 2) [34]. Furthermore, the behavior of enaminone **5** toward binucleophilic reagents was also investigated. Thus, when compound **5** was allowed to react with hydrazine hydrate a compound with a molecular formula C_20_H_14_N_2_O_2_ = 314 (M^+^) is formed which may be formulated as structure **14** based on spectroscopic data. Thus, the ^1^H-NMR spectrum of compound **14** showed a singlet signal at *δ* = 7.22 assigned to CH-pyrazole, a multiplet signals at *δ* = 7.31–8.71 assigned for CH aromatic and NH protons and a singlet signal at *δ* = 8.73 assigned to OH group. On the other hand, compound **6** was reacted with hydrazine hydrate to afford the product identical in all respects (mp, mixed mp, and spectral data) with those corresponding to the pyrazole derivative **14** as demonstrated in (Scheme 2).

**Scheme 3:**
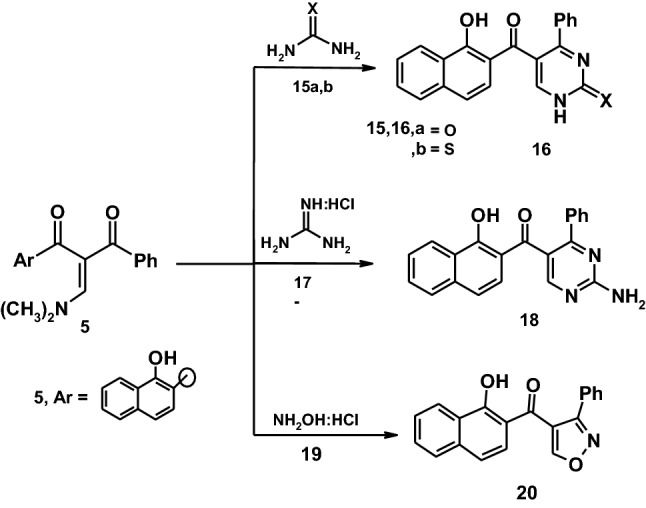
Synthetic route to pyrimidine and isoxazole

Similarly, reactions of enaminone **5** with urea or thiourea to yield the expected pyrimidine derivatives **16a,b**. Compounds **16a,b** were established on its correct spectral data (IR,^1^H-NMR, mass spectra and elemental analyses). Also, enaminone **5** reacted with guanidine hydrochloride to yield the pyrimidine derivative **18** which established on spectral data. On the other hand, the reaction of enaminone **5** with hydroxylamine hydrochloride in ethanol containing anhydrous sodium acetate afforded the isoxazole derivative **20**. The structure of compound **20** was established based on its elemental analyses and spectroscopic data. Thus, the ^1^H-NMR spectrum of compound **20** revealed the presence of a singlet signal at *δ* = 7.21 ppm corresponding to the CH-isoxazole and a multiplet signals at *δ* = 7.31–8.70 ppm corresponding to aromatic protons and a singlet signal at 8.71 ppm corresponding to OH group. The mass spectrum of compound **20** revealed a molecular ion peak at *m*/*z* = 315 (M^+^) corresponding to a molecular formula C_20_H_13_NO_3_ as demonstrated in (Scheme 3).

**Scheme 4:**
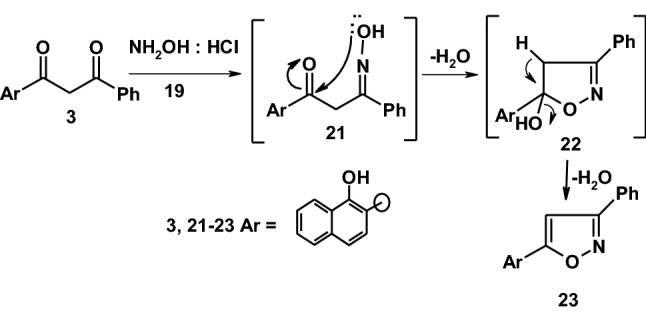
The synthesis route of compound **23**

3-Phenylpropane-1,3-dione derivative **3** was examined as a key precursor toward a variety of nucleophlic and electrophilic reagents aiming at exploring its synthetic potentiality. Thus, when compound **3** was reacted with hydroxylamine hydrochloride to yield the isoxazole derivative **23** in a good yield. Compound **23** was established based on its spectral data (IR, ^1^H-NMR, ^13^C-NMR, mass spectra) and elemental analyses. The ^1^H-NMR spectrum of compound **23** showed a singlet signal at *δ* = 7.21 ppm assigned to CH-isoxazole, a singlet signal at *δ* = 8.69 ppm assigned to OH group beside a multiplet signals at *δ* = 7.32–8.54 ppm assigned to aromatic protons. The mass spectrum showed a molecular ion peak at *m*/*z* = 287 (M^+^) corresponding to a molecular formula C_19_H_13_NO_2_ as demonstrated in (Scheme 4).

**Scheme 5:**
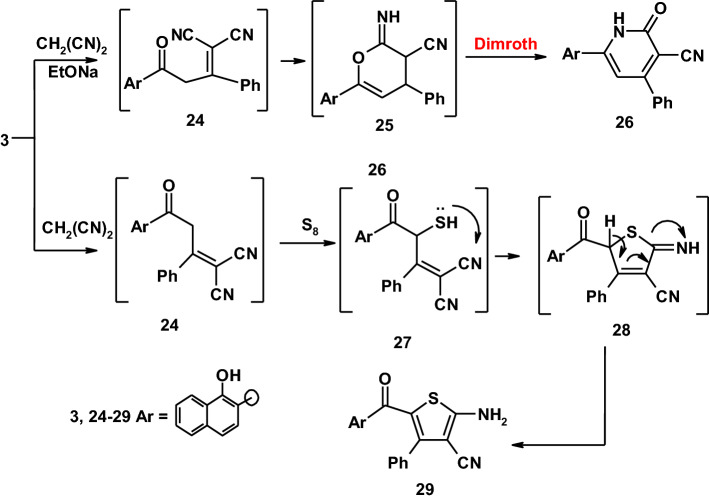
Synthetic route to pyridine and thiophene

The reactivity of compound **3** toward active methylene reagents was also investigated and found to afford new pyridinone derivative. Thus, when compound **3** was reacted with malononitrile in ethanolic sodium ethoxide, a product **26** with molecular formula C_22_H_14_N_2_O_2_ was obtained as the sole isolable product through the intermediates **24** and **25** [35]. On the other hand, the reaction of compound **3** with a mixture of malononitrile and elemental sulfur in ethanolic piperidine afforded the expected thiophene derivative **29** as demonstrated in (Scheme 5) [34]. Assignment of structure **29** for the reaction product was based on its correct elemental analyses and compatible spectroscopic data, the ^1^H-NMR of the structure revealed a singlet signal at *δ* = 8.69 ppm corresponding to OH group beside a multiplet signals corresponding to the aromatic protons and NH_2_ at *δ* = 7.20–8.68 ppm. Also, the mass spectrum showed a molecular ion peak at *m*/*z* = 370 (M^+^) corresponding to a molecular formula C_22_H_14_N_2_O_2_S.

**Scheme 6:**
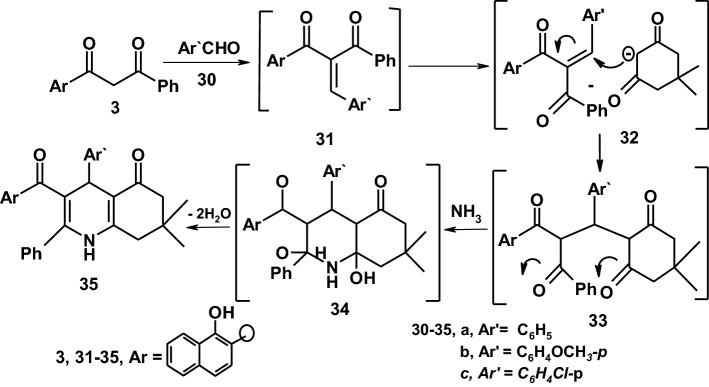
Synthetic route to fused pyridine

One-pot reaction of a mixture of compound **31** and dimedone in the presence of ammonium acetate afforded the tetrahydroquinoline derivatives **35a–c**. Assignment of structure **35b** as example for the reaction product was based on its compatible spectroscopic data. Thus, its IR spectrum showed an absorption bands at 3371, 3255 cm^−1^ for (OH/NH), 3059 cm^−1^ for (CH-arom), 2954–2835 cm^−1^ for (CH-aliph) and 1639, 1620 cm^−1^ for (2C=O) group. However, the ^1^H-NMR of compound **35b** for example showed a singlet signals at *δ* = 0.82, 1.19 ppm corresponding to 2CH_3_, two singlet signals at *δ* = 2.70 ppm and at *δ* = 2.86 ppm corresponding to two methylene groups of dimedone, a singlet signal at *δ* = 3.70 ppm assigned to OCH_3_ protons, a singlet signal at *δ* = 4.49 ppm assigned to CH-pyridine, a singlet signal at *δ* = 8.45 ppm assigned to OH group beside a multiplet signals at *δ* = 7.21–8.28 ppm assigned to aromatic protons and NH. The mass spectrum of the same compound revealed a molecular ion peak at 529 (M^+^) and a number of fragments which agree with the proposed structure. The formation of **35a–c** can be understood in terms of the Michael type addition of the active methylene group in the dimedone molecule to the activated double bond in the compound **31** to yield the Michael adduct **33** and cyclized to **34**, the intermediate **34** was oxidized by loss of water molecule to yield the final product **35** [36–38] as demonstrated in (Scheme 6).

**Scheme 7:**
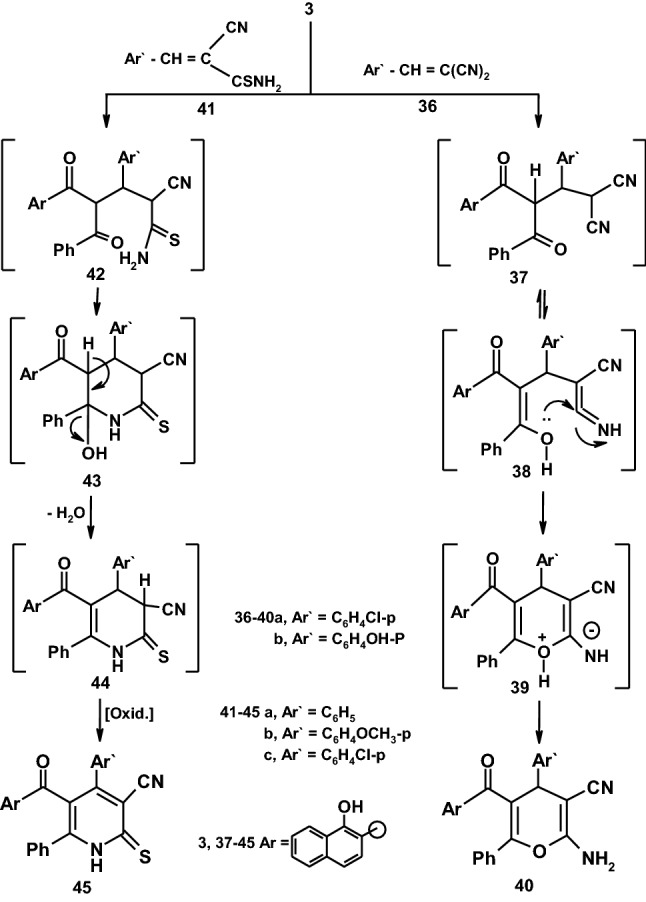
Reaction of 1,3-diketone with some electrophilic reagents

Reactions of compound **3** with some electrophilic reagents under alkaline conditions were also investigated. Thus, 4H-pyran-3-carbonitrile derivatives **40a,b** were synthesized in an excellent yield upon treatment of **3** with arylidinemalononitrile **36a–b** in the presence of a catalytic amount of piperidine. The structures of compounds **40a,b** were established based on analytical and spectral data.The products **40a,b** are apparently formed via a Michael type addition of the active methylene group in compound **3** to the activated double bond in arylidinemalononitrile **36a,b** to form the non-isolable intermediate **39** through an intramolecular cyclization and subsequent tautomerization to give **40a,b**. Also, compound **3** was reacted with arylidinecyanothioacetamide to yield the acceptable pyridinethione derivatives **45a–c**. We first assumed that the reaction product is formed via addition of active methylene to α, β-unsaturated linkage and subsequent water elimination from carbonyl benzoyl group to yield a cyclic intermediate **44** which aromatized by loss of H_2_ to yield the pyridinethione **45** as demonstrated in (Scheme 7). Confirmation of pyridinethiones **45** based on its compatible spectroscopic data. Thus, the ^1^H-NMR spectrum of compound **45b** for example showed a singlet signal at *δ* = 3.87 ppm assigned for OCH_3_, a singlet signal at *δ* = 8.74 ppm assigned to OH, a singlet signal at *δ* = 9.87 ppm assigned for NH group in addition to a multiplet signals at *δ* = 7.12–8.73 ppm assigned to aromatic protons [34, 39, 40].

**Scheme 8:**
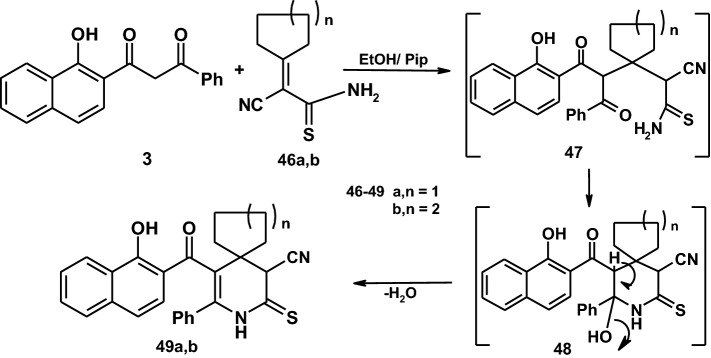
The synthetic route of compound **49**

Similarly, The reaction of compound **3** with cyclopentylidenecynothiocetamide and cyclohexyliden-cynothioacetamide **46a,b** yield the azaspiro derivatives **49a,b** through the intermediates **47**and **48** [41] as demonstrated in (Scheme 8). Establishing the spiro derivatives was based on its compatible spectroscopic data and elemental analyses.

**Scheme 9:**
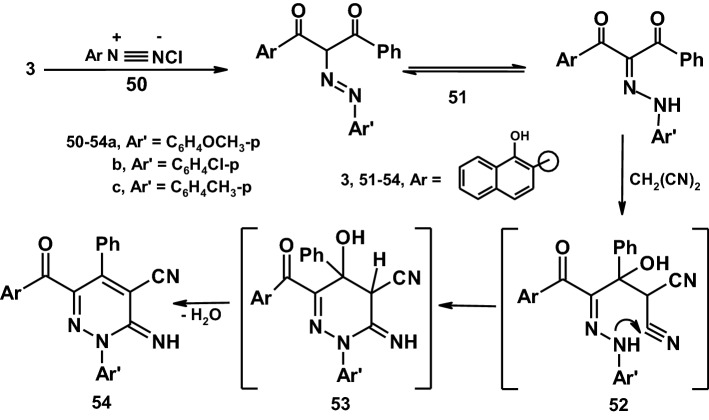
Synthetic route to pyridaznes

Coupling of propane-1,3-dione derivative **3** with diazotized aromatic amines in ethanol buffered with sodium acetate at 0–5 °C afforded the aryl hydrazones **51a–c**. The aryl hydrazones were established based on its compatible spectroscopic data and elemental analyses. Condensation of **51** with malononitrile proceeded very readily by fusion in the presence of ammonium acetate to yield the pyridazine derivatives **54 a–c**. Compounds **54a–c** were established based on spectral data (IR, ^1^H-NMR and mass spectra) and elemental analyses. Thus, the ^1^H-NMR spectrum of **54a** for example revealed a singlet signal at *δ* = 3.99 ppm assigned to OCH_3_, a singlet signal at *δ* = 8.71 ppm assigned to OH group in addition to a multiplet signals at *δ* = 6.68–8.25 ppm corresponding to aromatic protons and NH. Also, the mass spectrum of the same compound revealed a molecular ion peak at 472 (M^+^) corresponding to the molecular formula C_29_H_20_N_4_O_3_ and a number of fragments which agree with the proposed structure [42] (Scheme 9).

## In vitro antimicrobial activity

The newly synthesized compounds have been screened for antibacterial activity against some species of Gram-positive bacteria *(Staphylococcus aureus* and *Bacillus subtilis)* and Gram-negative bacteria (*Escherichia coli* and *Pseudomonas aeuroginosa)*. Anti-fungal activities of the compounds were tested against yeast and mycelial fungi; *Candida albicans* and *Aspergillus flavus*, respectively. Each tested compound was dissolved in DMSO making a solution concentration of 1.00 mg/mL and loaded separately in paper disks of Whatman filter paper with equal diameter size (10 mm), Paper disks were sterilized in an autoclave. The paper disks loaded with the desired concentration of the complex solution, were placed aseptically in the petri dishes containing nutrient agar medium (agar 20 g + beef extract 3 g + peptone 5 g) inoculated with *Staphylococcus aureus, Bacillus subtilis, Escherichia coli, Pseudomonas aeuroginosa, Candida albicans* and *Aspergillus flavus*. The petri dishes were incubated at 36 °C. The inhibition zones were recorded after 24 h of incubation in case of bacteria and yeast and after 5–6 days in case of mycelial fungi. Each treatment was replicated three times [43, 44]. *Ampicillin* and *clotrimazole*, were used as a common standard antibiotic and antifungal agents, respectively. They prepared using the same procedure as above at the same concentration and solvents. The % activity index was calculated for the tested compounds by using the given formula in Eq. (1).

1 $$ {\text{\% Activity index = }}\frac{{\text{Zone of inhibition by test compound (diameter)}}}{{\text{Zone of inhibition by standard (diameter)}}} \times 100 $$

## Minimum inhibitory concentration (MIC) measurement

The minimum inhibitory concentration (MIC) was determined using the disk diffusion technique by preparing disks containing 1.9–1000 µg/mL of each compound against Gram-positive bacteria (*Staphylococcus aureus, Bacillus subtilis)* and Gram-negative bacteria (*Escherichia coli, Pseudomonas aeuroginosa)*. The anti-fungal activities of the compounds were tested against two fungi *Candida albicans and Aspergillus flavus*. The twofold dilutions of the solution were prepared. The microorganism suspensions at 10 CFU/mL (colony forming unit/mL) concentration were inoculated to the corresponding wells. The plates were incubated at 36 °C for 24 h for the bacteria. The standard antibiotic *ampicillin* and antifungal *clotrimazole* was also recorded using the same procedure as above at the same concentration and solvents. At the end of the incubation period, the minimum inhibitory concentrations (MIC) values were recorded as the lowest concentration of the substance that had no visible turbidity. Control experiments with DMSO and uninoculated media were run parallel to the test compounds under the same condition. Tables 1 and 2 illustrated the results of antimicrobial and antifungal activity and it's MIC. The results which are illustrated in Table 1 showed that most of tasted compounds were active against most of micro-organisms used. Both of compounds **45c** and **5** showed no antibacterial or antifungal activity. On the other side each of compound **29** and **54a** showed maximum antibacterial and antifungal activity. Compound **51a** has no antibacterial activity against Gram-negative bacteria only, although it have broad spectrum antibacterial activity against Gram-positive bacteria and antifungal activity against *C. albicans* and *A. flavus*. On the other hands, compound **23** showed narrow spectrum antibacterial activity against *P. aeuroginosa* (a Gram-negative bacteria) and *S. aureus* (a Gram-positive bacteria) and revealed no antibacterial activity against *E. coli* (a Gram-negative bacteria) and *B. subtilis* (a Gram-positive bacteria), but in case of compound **40b** it has no antibacterial activity against *B. Subtilis* only and has narrow range spectrum as antibacterial agent against *S. aureus, E. Coli* and *P. aeuroginosa* with also small rang spectrum antifungal activity. All the other compounds **(3, 35b, 26, 35c, 35a, 40a, 10, 16a, 14,and 49a)** indicated wide range spectrum antibacterial and antifungal activity.

**Table 1:** Antibacterial and antifungal activities of synthesized compounds

Compound	Gram-negative bacteria (−ve)				Gram-positive bacteria (+ve)				Fungal Species			
	*E. coli*		*P. aeuroginosa*		*S. aureus*		*B. subtilis*		*C. albicans*		*A. flavus*	
	DIZ (mm)	% Activity index	DIZ (mm)	% Activity index	DIZ (mm)	% Activity index	DIZ (mm)	% Activity index	DIZ (mm)	% Activity index	DIZ (mm)	% Activity index
**35c**	4.1	13.7	9	33.5	13.4	52.1	12	45.6	13	44.6	15	57.0
**40a**	5.2	18.8	13	44.5	14.7	56.8	14.3	54.4	16.3	55.2	17.4	66.6
**51a**	NA	–	NA	–	10	39.6	8	32.4	13	45.7	14	49.2
**29**	18.4	79.9	23	94.5	25	93.5	24.8	98.9	19.1	77.3	24	88.1
**10**	15	64.6	17.5	70.9	22.5	86.3	19	87.9	19.3	73.8	24.8	83.7
**40b**	3	10.5	7	23.8	5	14.7	NA	–	4	13.5	6	24.0
**14**	8	29.6	10	48.7	9	26.7	6	18.9	8	25.2	13	43.4
**16a**	14.3	54.5	15	66.7	16.7	80.0	19.6	85.6	19	59.7	17.8	69.1
**3**	9	37.8	19	61.5	14	46.3	11	36.8	11	22.8	10	33.0
**35a**	15	70.0	20	83.9	19.7	73.8	19.6	77.3	17.6	63.5	19.4	73.6
**35b**	11	40.8	17.3	63.9	16.9	71.7	17.8	69.4	12.9	56.0	18	62.0
**23**	NA	–	4	13.8	3	8.9	NA	–	5	12.3	7	17.0
**26**	13	49.5	16	69.2	14	65.5	14	58.5	10	25.6	14	42.0
**45c**	NA	–	NA	–	NA	–	NA	–	NA	–	NA	–
**49a**	5	24.8	12	54.2	8	35.3	6	21.7	8	33.3	11	44.0
**5**	NA	–	NA	–	NA	–	NA	–	NA	–	NA	–
**54a**	11	77.2	27	83.9	24	84.5	31	97.6	24.7	85.3	28.5	97.0
Ampicillin	25	100	25	100	24	100	25	100	NA	–	NA	–
Clotrimazole	NA	–	NA	–	NA	–	NA	–	26	100	26	100

*NA* No activity, *DIZ* Diameter of inhibition zone

**Table 2:** Minimum inhibitory concentrations (MIC) for selected compounds

Compounds	Minimum inhibitory concentration (MIC) of the synthesized compounds (µg/mL)					
	*E. coli*	*P. aeuroginosa*	*S. aureus*	*B. subtilis*	*C. albicans*	*A. flavus*
**35c**	760	530	270	500	65.5	50.9
**40a**	780	385	260	500	48.9	29.4
**51a**	NA	NA	530	740	95.7	69.5
**29**	98.7	68.5	66.5	135	14.6	8.8
**10**	197.5	135	90.7	189.4	22.4	9.8
**40b**	NA	750	NA	NA	510	365
**14**	540	260	740	NA	188.5	93.7
**16a**	188.4	125	125	177.5	37.2	17.6
**3**	385	187.5	375	740	260	187.5
**35a**	145	92.7	135.7	240	24.4	14.7
**35b**	365	197.5	177.9	240	63.5	39.2
**23**	NA	NA	NA	NA	760	600
**26**	240	136	187.8	385	260	145
**45c**	NA	NA	NA	NA	NA	NA
**49a**	520	260	500	NA	125	97.7
**5**	NA	NA	NA	NA	NA	NA
**54a**	125	93.7	93.7	125	11.7	3.9
Ampicillin	125	187.5	93.7	187.5	NA	NA
Clotrimazole	NA	NA	NA	NA	7.8	5.8

*NA* No activity

From Table 2, we observed that compounds **29, 35a, 10, 54a and 16a** showed the lowest minimum inhibitory concentrations (MIC) for most tested bacteria and fungi, while compounds **40a, 40b, 35c** and **49a** exhibited high concentrations of MIC as compared with standard antimicrobial agents used.

Structure–activity relationship: By analyzing the previous results, it is noted that the substitutes do not play a clear role in the biological activity. However, in most of the results it was observed that the compounds that contain electron-withdrawing groups have a higher biological activity than the compounds that contain electron-donating groups and that the biological activity depends on the formation of the new fused rings and the type of strains chosen from bacteria and fungi.

## Conclusion

In conclusion, compounds **3** and **5** were used as efficient precursors for the synthesis of new heterocycles including α-naphthol moiety with expected biological activities.

## Experimental

The melting points, the elemental analyses and the spectral data were recorded as reported in reference [29].

### General procedure of the synthesis of 1-(1-hydroxynaphthalen-2-yl)-3-phenyl-propane-1,3-dione (3)

A mixture of α-naphthol **1** (0.01 mol, 1.44 g), ethyl benzoylacetate **2** (0.01 mol, 1.92 g) and zinc chloride (0.5 gm) was exposed to microwave irradiation (Microwave assisted synthesis was performed on a CEM Microwave synthesizer, the irradiation power was 200 W as the maximum level of irradiation and a maximum level of internal vessel pressure at 250 Psi for about 5 min), the reaction mixture was allowed to reach room temperature, then diluted with ethanol with stirring and the solid product that formed, was filtrated off and crystallized from ethanol to give (**3**) as brown crystals: M.P.: 147–149 °C, yield: 2.65 g (91%). IR (KBr) (*v*_max_/cm^−1^): 3365 (OH), 3061 (Ar–H), 2924 (Aliph-H), 1720, 1639 (2C=O). MS (EI, 70 eV): *m*/*z* (%) = 290 (M^+^). ^1^H NMR (400 MHz, DMSO-d_6_): *δ* (ppm) 4.49 (s, 2H, CH_2_), 7.20–8.68 (m, 11H, aromatic H), 8.69 (s, 1H, OH). Anal. Calcd. for C_19_H_14_O_3_ (290): C, 78.61; H, 4.86. Found: C, 78.63; H, 4.88.

#### Synthesis of 2-((dimethylamino)methylene)-1-(1-hydroxynaphthalen-2-yl)-3-phenyl-propane-1,3-dione (**5**)

A mixture of **3** (0.01 mol, 2.9 g) and DMF-DMA (0.01 mol, 1.19 g) in dioxane (30 ml) was heated under reflux for 6 h. The reaction mixture was allowed to cool. The separated solid was filtered off, washed with ethanol and crystallized from ethanol to give (**5**) as pale brown crystals: M.P.: 173–175 °C, yield: 2.65 g (77%). IR (KBr) (*v*_max_/cm^−1^): 3422 (NH), 3062 (Ar–H), 2932–2854 (Aliph- H), 1723, 1636 (2C=O). MS (EI, 70 eV): *m*/*z* (%) = 345 (M^+^). ^1^H NMR (400 MHz, DMSO-d_6_): *δ* 3.00 (s, 6H, 2CH_3_), 7.19 (s, 1H, CH-oleffinic), 7.64–8.67 (m, 11H, aromatic H), 8.68 (s, 1H, OH). ^13^C NMR (100 MHz, DMSO-d_6_): *δ* (ppm) 44.5, 44.5, 116.2, 122.4, 124.3, 126.1, 126.7, 127.6, 127.6, 128.1, 128.1, 128.2, 128.6, 129.1, 130.1, 132.4, 132.5, 138.1, 163.2, 166.5, 194.7, 198.0. Anal. calcd for C_22_H_19_NO_3_ (345): C, 76.50; H, 5.54; N, 4.06. Found: C, 76.52; H, 5.56; N, 4.08.

#### Synthesis of 2-(ethoxymethylene)-1-(1-hydroxynaphthalen-2-yl)-3-phenylpropane-1,3-dione (**6**)

A mixture of **3** (0.01 mol, 2.9 g) and triethoxy methane (3 ml) in acetic anhydride (10 ml) was heated under reflux for 12 h. The reaction mixture was allowed to cool. The separated solid was filtered off, washed with ethanol and crystallized from ethanol to give (**6**) as pale brown crystals: M.P.: 120–122 °C, yield: 3.46 g (83%). IR (KBr) (*v*_max_/cm^−1^): 3426 (OH), 3062 (Ar–H), 2930 (Aliph-H), 1767, 1636 (2C=O). MS (EI, 70 eV): *m*/*z* (%) = 346 (M^+^). ^1^H NMR (400 MHz, DMSO-d_6_): *δ* (ppm) 1.11 (t, 3H, CH_3_), 4.19 (q, 2H, CH_2_), 6.33 (s, 1H, CH-oleffinic), 7.29–8.23 (m, 11H, aromatic H), 8.68 (s, 1H, OH). Anal. calcd for C_22_H_18_O_4_ (346): C, 76.29; H, 5.24. Found: C, 76.31; H, 5.26.

#### Synthesis of 5-(1-hydroxy-2-naphthoyl)-4-phenyl-2-thioxo-1,2-dihydropyridine-3-carbonitrile (**10**)

A mixture of **5** (0.01 mol, 3.45 g) and cyanothioacetamide **7** (0.01 mol, 1 g), in presence of sodium ethoxide (30 ml) was heated under reflux for 24 h. The solution was allowed to cool and poured into crushed ice then acidified with HCl. The separated solid was filtered off, washed with water and crystallized from dioxane to give (**10**) as brown crystals: M.P.: 238–240 °C, yield: 3.35 g (87%). IR (KBr) (*v*_max_/cm^−1^): 3447, 3423 (NH), 3062 (AR–H), 2209 (CN), 1636 (C=O).). MS (EI, 70 eV): *m*/*z* (%) = 382 (M^+^). ^1^H NMR (400 MHz, DMSO-d_6_): *δ* (ppm) 7.20–8.24 (m, 13H, aromatic H and NH), 8.70 (s, 1H, OH). ^13^C NMR (100 MHz, DMSO-d_6_): *δ* (ppm) 107.1, 117.0, 118.2, 122.3, 123.2, 126.3, 126.4, 126.8, 127.3, 127.3, 127.5, 127.5, 128.3, 128.4, 129.1, 130.0, 130.1, 132.2, 144.6, 166.1, 168.0, 168.6, 196.0. Anal. calcd for C_23_H_14_N_2_O_2_S (382): C, 72.23; H, 3.69; N, 7.33. Found: C, 72.25; H, 3.71; N, 7.35.

#### Synthesis of (1-hydroxynaphthalen-2-yl)(3-phenyl-1H-pyrazol-4-yl)methanone (**14**)

Method (A): A mixture of **5** (0.01 mol, 3.45 g) and hydrazine hydrate (10 ml) was heated under reflux for 12 h. The reaction mixture was allowed to cool and poured into crushed ice. The separated solid was filtered off, washed with water and crystallized from DMF to give (**14**).

Method (B): A mixture of **6** (0.01 mol) and hydrazine hydrate (10 ml) was heated under reflux for 12 h. The reaction mixture was allowed to cool and poured into crushed ice. The separated solid was filtered off, washed with water and crystallized from DMF to give (**14**).

as white crystals: M.P.: > 300 °C, yield: 2.50 g (79%). IR (KBr) (*v*_max_/cm^−1^): 3300, 3228 (NH), 3062 (Ar–H), 1670 (C=O).). MS (EI, 70 eV): *m*/*z* (%) = 314 (M^+^). ^1^H NMR (400 MHz, DMSO-d_6_): *δ* (ppm) 7.22 (s, 1H, CH-pyrazole), 7.31–8.71 (m, 12H, aromatic and NH), 8.73 (s, 1H, OH). Anal. calcd for C_20_H_14_N_2_O_2_ (314): C, 76.42; H, 4.49; N, 8.91. Found: C, 76.44; H, 4.51; N, 8.93.

### General procedure for preparation of compounds (16a,b and 18)

A mixture of **5** (0.01 mol, 3.45 g) with urea (0.01 mol, 0.6 g), thiourea (0.01 mol, 0.76 g) and guanidine hydrochloride (0.01 mol, 0.95 g), in presence of sodium ethoxide (30 ml) was heated under reflux for 24 h. The solutions were allowed to cool and poured into crushed ice then acidified with HCl. The separated solids were filtered off, washed with water and crystallized from the proper solvent to give (**16a,b** and **18**).

#### 5-(1-hydroxy-2-naphthoyl)-4-phenylpyrimidin-2(1H)-one (**16a**)

It was obtained as beige crystals from DMF: M.P.: > 300 °C, yield: 2.7 g (78%). IR (KBr) (*v*_max_/cm^−1^): 3447 (OH), 3423 (NH), 3061 (Ar–H), 1740, 1641 (2C=O). MS (EI, 70 eV): *m*/*z* (%) = 342 (M^+^). ^1^H NMR (400 MHz, DMSO-d_6_): *δ* (ppm) 7.22 (s, 1H, CH-pyrimidine), 7.65–8.70 (m, 12H, aromatic H and NH), 8.71 (s, 1H, OH). Anal. calcd for C_21_H_14_N_2_O_3_ (342): C, 73.68; H, 4.12; N, 8.18. Found: C,73.70; H, 4.14; N,8.20.

#### (1-hydroxynaphthalen-2-yl)(4-phenyl-2-thioxo-1,2-dihydropyrimidin-5-yl)methanone (**16b**)

It was obtained as brown crystals from DMF: M.P.: > 300 °C, yield: 2.8 g (78%). IR (KBr) (*v*_max_/cm^−1^): 3449 (OH), 3400 (NH), 1645 (C=O). MS (EI, 70 eV): *m*/*z* (%) = 358 (M^+^). ^1^H NMR (400 MHz, DMSO-d_6_): *δ* (ppm) 7.19 (s, 1H, CH-pyrimidine), 7.59–8.67 (m, 12H, aromatic H and NH), 8.68 (s, 1H, OH). Anal. calcd for C_21_H_14_N_2_O_2_S (358): C, 70.37; H, 3.94; N, 7.82. Found: C, 70.39; H, 3.96; N, 7.84.

#### (2-amino-4-phenylpyrimidin-5-yl)(1-hydroxynaphthalen-2-yl)methanone (**18**)

It was obtained as brown crystals from dioxane: M.P.: 250–252 °C, yield: 2.45 g (71%). IR (KBr) (*v*_max_/cm^−1^): 3449–3400 (OH, NH_2_), 1642 (C=O). MS (EI, 70 eV): *m*/*z* (%) = 341 (M^+^). ^1^H NMR (400 MHz, DMSO-d_6_): *δ* (ppm) 6.20 (s, 2H, NH_2_), 7.31–8.39 (m, 12H, aromatic H), 8.67 (s, 1H, OH). ^13^C NMR (100 MHz, DMSO-d_6_): *δ* (ppm) 118.0, 119.3, 123.2, 124.3, 124.8, 126.4, 126.4, 126.4, 126.6, 127.6, 128.2, 128.7, 128.9, 128.9, 130.7, 132.7, 155.3, 162.7, 165.8, 166.3, 194.0. Anal. calcd for C_21_H_15_N_3_O_2_ (341). Anal. Calcd. for C_21_H_15_N_3_O_2_: C, 73.89; H, 4.43; N, 12.31. Found: C, 73.91; H, 4.45; N, 12.33.

#### Synthesis of (1-hydroxynaphthalen-2-yl)(3-phenylisoxazol-4-yl)methanone (**20**)

A mixture of **5** (0.01 mol, 3.45 g), hydroxylamine hydrochloride in ethanol (30 ml) containing anhydrous sodium acetate (1 g) was heated under reflux for 24 h. The reaction mixture was allowed to cool and poured into cold water (60 ml). The separated solid was filtered off and crystallized from ethanol to give (**20**) as brown crystals: M.P.: 150–152 °C, yield: 2.25 g (71%). IR (KBr) (*v*_max_/cm^−1^): 3425 (OH), 3060 (Ar–H), 1636 (C=O). MS (EI, 70 eV): *m*/*z* (%) = 315 (M^+^). ^1^H NMR (400 MHz, DMSO-d_6_): *δ* (ppm) 7.21 (s, 1H, CH-isoxazole), 7.31–8.70 (m, 11H, aromatic H), 8.71 (s, 1H, OH). Anal. calcd for C_20_H_13_NO_3_ (315): C, 76.18; H, 4.16; N, 4.44%. Found:C, 76.20; H, 4.18; N, 4.46%.

#### Synthesis of (2-(3-Phenylisoxazol-5-yl)naphthalen-1-ol (**23**)

A mixture of **3** (0.01 mol), hydroxylamine hydrochloride (0.01 mol) in ethanol (30 ml) containing anhydrous sodium acetate (1 g) was heated under reflux for 24 h. The reaction mixture was allowed to cool and poured into cold water (60 ml). The separated solid was filtered off and crystallized from ethanol to give (**23**) as beige crystals: M.P.: 132–134 °C, yield: 2.25 g (80%). IR (KBr) (*v*_max_/cm^−1^): 3382 (NH), 3061 (Ar–H). MS (EI, 70 eV): *m*/*z* (%) = 287 (M^+^). ^1^H NMR (400 MHz, DMSO-d_6_): *δ* (ppm) 7.21 (s, 1H, CH- isoxazole), 7.32–8.54 (m, 11H, aromatic H), 8.69 (s, 1H, OH); ^13^C NMR (100 MHz, DMSO-d_6_) *δ* 99.6, 118.7, 120.6, 123.5, 124.1, 126.1, 126.3, 126.5, 126.5, 127.3, 127.5, 127.6, 127.9, 128.8, 128.8, 132.4, 161.0, 163.2, 170.1. Anal. calcd for C_19_H_13_NO_2_ (287): C, 79.43; H, 4.56; N, 4.88. Found: C, 79.45; H, 4.58; N, 4.90.

#### Synthesis of 6-(1-hydroxynaphthalen-2-yl)-2-oxo-4-phenyl-1,2-dihydropyridine-3-carbonitrile (**26**)

A mixture of **3** (0.01 mol, 2.90 g), malononitrile (0.01 mol, 0.66 g) in sodium ethoxide (30 ml) was heated under reflux for 12 h. The reaction mixture was allowed to cool and poured into crushed ice then acidified with HCl. The separated solid was filtered off, washed with water and crystallized from ethanol to give (**26**) as brown crystals: M.P.: 163–165 °C, yield: 2.35 g (69%). IR (KBr) (*v*_max_/cm^−1^): 3311, 3294, 3062, 2193, 1639 cm^−1^. MS (EI, 70 eV): *m*/*z* (%) = 338 (M^+^). ^1^H NMR (400 MHz, DMSO-d_6_): *δ* (ppm) 7.16 (s, 1H, CH-Pyridine), 7.51–8.63 (m, 12H, aromatic H and NH), 8.64 (s, 1H, OH). ^13^C NMR (100 MHz, DMSO-d_6_): *δ* (ppm) 108.3, 118.3, 118.6, 120.1, 123.4, 124.1, 125.8, 126.9, 127.1, 127.2, 127.6, 127.6, 127.9, 127.9, 128.3, 133.9, 135.2, 153.6, 155.2, 155.3, 157.8, 162.3. Anal. calcd for C_22_H_14_N_2_O_2_ (338): C, 78.09; H, 4.17; N, 8.28. Found: C, 78.11; H, 4.19; N, 8.30.

#### Synthesis of 2-amino-5-(1-hydroxy-2-naphthoyl)-4-phenylthiophene-3-carbonitrile (**29**)

Equimolar amounts of **3** (0.01 mol, 2.90 g), malononitrile and elemental sulfur (0.01 mol, 0.66 and 0.32 g) in ethanol (30 ml) containing piperidine were refluxed for 15 h, poured onto cold water (60 ml) and acidified with HCl. The solid product thus formed was filtered off and crystallized from ethanol to give (**29**) as yellow crtstals: M.P.: 130–132 °C, yield: 2.95 g (80%). IR (KBr) (*v*_max_/cm^−1^): 3421–3400 (NH_2_), 3061 (Ar–H), 2192 (CN), 1639 (C=O). MS (EI, 70 eV): *m*/*z* (%) = 370 (M^+^). ^1^H NMR (400 MHz, DMSO-d_6_): *δ* (ppm) 7.20–8.68 (m, 13H, aromatic H and NH_2_), 8.69 (s, 1H, OH). Anal. calcd for C_22_H_14_N_2_O_2_S (370): C, 71.33; H, 3.81; N, 7.56. Found:C, 71.35; H, 3.84; N, 7.58.

### General procedure for preparation of compounds (35a-c)

A mixture of **31a–c** (which prepared by a mixture of **3** and **30a–c** in ethanol/piperidine/ refluxing) (0.01 mol, 2.90 g), dimedone (0.01 mol, 1.40 g) and ammonium acetate (2 gm) was fused for 30 min. The reaction mixture was allowed to cool, then triturated with ethanol. The separated solid was filtered off, washed with water and crystallized from the proper solvent to give (**35a–c)**.

#### 3-(1-hydroxy-2-naphthoyl)-7,7-dimethyl-2,4-diphenyl-4,6,7,8-tetrahydroquinolin-5(1H)-one (**35a**)

It was obtained as yellow crystals from dioxane: M.P.: 280–282 °C, yield: 4.30 g (86%). IR (KBr) (*v*_max_/cm^−1^): 3402, 3400 (NH), 3059 (Ar–H), 2954–2870 (Aliph-H), 1642, 1620 (2C=O). MS (EI, 70 eV): *m*/*z* (%) = 499 (M^+^). ^1^H NMR(400 MHz, DMSO-d_6_): *δ* (ppm) 0.86 (s, 3H, CH_3_), 0.99 (s, 3H, CH_3_), 2.73 (s, 2H, CH_2_), 2.89 (s, 2H, CH_2_), 4.80 (s, 1H, CH-pyridine), 7.14–8.70 (m, 17H, aromatic H and NH), 8.70 (s, 1H, OH). Anal. calcd for C_34_H_29_NO_3_ (499): C, 81.74; H, 5.85; N, 2.80. Found: C, 81.76; H, 5.87; N, 2.82.

#### 3-(1-hydroxy-2-naphthoyl)-4-(4-methoxyphenyl)-7,7-dimethyl-2-phenyl-4,6,7,8-tetrahydro-quinolin-5(1H)-one (**35b**)

It was obtained as yellow crystals from dioxane: M.P.: 277–279 °C, yield: 4.25 g (80%). IR (KBr) (*v*_max_/cm^−1^): 3371 (OH), 3255 (NH), 3059 (Ar–H), 2954–2835 (Aliph-H), 1639, 1620 (2C=O). MS (EI, 70 eV): *m*/*z* (%) = 529 (M^+^). ^1^H NMR (400 MHz, DMSO-d_6_): *δ* (ppm) 0.82(s, 3H, CH_3_), 1.19 (s, 3H, CH_3_), 2.70 (s, 2H, CH_2_), 2.86 (s, 2H, CH_2_), 3.70 (s, 3H, OCH_3_), 4.49 (s, 1H, CH-pyridine), 7.21–8.28 (m, 16H, aromatic H and NH), 8.45 (s, 1H, OH). ^13^C NMR (100 MHz, DMSO-d_6_): *δ* (ppm) 29.2, 29.8, 33.8, 41.0, 41.4, 52.3, 57.8, 107.0, 113.2, 116.3, 116.3, 122.0, 123.1, 124.3, 126.4, 126.8, 127.2, 127.6, 127.6, 127.6, 127.8, 128.0, 128.2, 128.9, 132.1, 132.1, 132.7, 137.2, 137.2, 143.6, 150.2, 159.5, 166.0, 193.4, 195.1. Anal. calcd for C_35_H_31_NO_4_ (529): C, 79.37; H, 5.90; N, 2.64. Found: C, 79.39; H, 5.92; N, 2.66.

#### 4-(4-chlorophenyl)-3-(1-hydroxy-2-naphthoyl)-7,7-dimethyl-2-phenyl-4,6,7,8-tetra-hydroquinolin-5(1H)-one (**35c**)

It was obtained as pale yellow crystals from dioxane: M.P.: 290–292 °C, yield: 4.30 g (83%). IR (KBr) (*v*_max_/cm^−1^): 3356 (OH), 3271 (NH), 3059 (Ar–H), 2954–2870 (Aliph-H), 1643, 1620 (2C=O). MS (EI, 70 eV): *m*/*z* (%) = 533 (M^+^). ^1^H NMR (400 MHz, DMSO-d_6_): *δ* (ppm) 0.83 (s, 3H, CH_3_), 1.20 (s, 3H, CH_3_), 2.70 (s, 2H, CH_2_), 2.89 (s, 2H, CH_2_), 4.49 (s, 1H, CH-pyridine), 7.28–8.11 (m, 16H, aromatic H and NH), 8.50 (s, 1H, OH). Anal. calcd for C_34_H_28_ClNO_3_ (533): C, 76.47; H, 5.28; N, 2.62. Found:C, 76.49; H, 5.30; N, 2.64.

### General procedure for preparation of compounds (40a,b)

A mixture of **3** (0.01 mol, 2.90 g) and arylidenemalononitriles **(36a,b)** (0.01 mol, 1.88 and 1.70 g) in ethanol (30 ml) containing a catalytic amount of piperidine was heated under reflux for 12 h. The reaction mixture was allowed to cool and poured into crushed ice then acidified with HCl. The separated solid was filtered off, washed with water and crystallized from the proper solvent to give **(40a,b).**

#### 2-amino-4-(4-chlorophenyl)-5-(1-hydroxy-2-naphthoyl)-6-phenyl-4H-pyran-3-carbonitrile (**40a**)

It was obtained as brown crystals from ethanol: M.P.: 162–164 °C, yield: 4.30 g (92%). IR (KBr) (*v*_max_/cm^−1^): 3448- 3421 (OH, NH_2_), 3063 (Ar–H), 2929 (Aliph-H), 2191 (CN), 1630 (C=O). MS (EI, 70 eV): *m*/*z* (%) = 480 (M^+^ + 2). ^1^H NMR (400 MHz, DMSO-d_6_): *δ* (ppm) 4.40 (s, 1H, 4H-pyrane), 7.23–8.72 (m, 17H, aromatic H and NH_2_), 8.74 (s, 1H, OH). ^13^C NMR (100 MHz, DMSO-d_6_): *δ* (ppm) 43.7, 60.3, 118.9, 122.1, 123.4, 124.2, 125.2, 125.2, 126.2, 126.8, 126.8, 126.8, 126.9, 126.9, 127.5, 127.5, 127.6, 127.6, 128.1, 128.2, 131.3, 131.3, 132.6, 133.2, 135.0, 140.8, 163.3, 165.2, 193.5. Anal. calcd for C_29_H_19_ClN_2_O_3_ (478): C, 72.73; H, 4.00; N, 5.85. Found: C, 72.75; H, 4.02; N, 5.87.

#### 2-amino-5-(1-hydroxy-2-naphthoyl)-4-(4-hydroxyphenyl)-6-phenyl-4H-pyran-3-carbonitrile (**40b**)

It was obtained as brown crystals from ethanol: M.P.: 200–202 °C, yield: 4.30 g (90%). IR (KBr) (*v*_max_/cm^−1^): 3447–3390 (OH, NH_2_), 3062 (Ar–H), 2928 (Aliph-H), 2196 (CN), 1630 (C=O). MS (EI, 70 eV): *m*/*z* (%) = 460 (M^+^). ^1^H NMR (400 MHz, DMSO-d_6_): *δ* (ppm) 4.20 (s, 1H, 4H-pyrane), 6.87 (s, 2H, NH_2_), 6.90–8.71 (m, 15H, aromatic H), 8.72 (s, 1H, OH), 10.00 (s, 1H, OH). Anal. calcd for C_29_H_20_N_2_O_4_ (460): C, 75.64; H, 4.38; N, 6.08. Found: C, 75.66; H, 4.40; N, 6.10.

### General procedure for preparation of compounds (45a–c)

A mixture of **3** (0.01 mol, 2.90 g) and arylidenecyanothioacetamide **(41a–c)** (0.01 mol, 1.88, 2.18 and 2.22 g) in ethanol (30 ml) containing a catalytic amount of TEA was heated under reflux for 12 h. The reaction mixture was allowed to cool and poured into crushed ice then acidified with HCl. The separated solid was filtered off, washed with water and crystallized from the proper solvent to give **(45a–c)**.

#### 5-(1-hydroxy-2-naphthoyl)-4,6-diphenyl-2-thioxo-1,2-dihydropyridine-3-carbonitrile (**45a**)

It was obtained as pale yellow crystals from ethanol: M.P.: 146–148 °C, yield: 3.60 g (78%). IR (KBr) (*v*_max_/cm^−1^): 3444 (OH), 3390 (NH), 3059 (Ar–H), 2191 (CN), 1620 (C=O). MS (EI, 70 eV): *m*/*z* (%) = 458 (M^+^). ^1^H NMR (400 MHz, DMSO-d_6_): *δ* (ppm) 7.23–8.73 (m, 17H, aromatic H and NH), 8.74 (s, 1H, OH). Anal. calcd for C_29_H_18_N_2_O_2_S (458): C, 75.96; H, 3.96; N, 6.11. Found: C, 75.98; H, 3.98; N, 6.13.

#### 5-(1-hydroxy-2-naphthoyl)-4-(4-methoxyphenyl)-6-phenyl-2-thioxo-1,2-dihydro-pyridine-3-carbonitrile (**45b**)

It was obtained as pale yellow crystals from ethanol: M.P.: 150–152 °C, yield: 4.00 g (82%). IR (KBr) (*v*_max_/cm^−1^): 3448, 3387, 3059, 2931, 2194, 1630 cm^−1^. MS (EI, 70 eV): *m*/*z* (%) = 488 (M^+^). ^1^H NMR (400 MHz, DMSO-d_6_): *δ* (ppm) 3.87 (s, 3H, OCH_3_), 7.12–8.73 (m, 15H, aromatic H), 8.74 (s, 1H, OH), 9.87 (s, 1H, NH). ^13^C NMR (100 MHz, DMSO-d_6_): *δ* (ppm) 57.6, 108.2, 111.9, 118.2, 118.2, 118.8, 122.3, 124.6, 124.8, 125.2, 126.4, 126.8, 126.8, 127.3, 127.3, 127.5, 127.6, 128.0, 128.1, 128.7, 132.1, 132.1, 132.7, 135.2, 155.8, 160.8, 163.7, 165.2, 172.6, 193.3. Anal. calcd for C_30_H_20_N_2_O_3_S (488): C, 73.75; H, 4.13; N, 5.73. Found: C, 73.77; H, 4.15; N, 5.75.

#### 4-(4-chlorophenyl)-5-(1-hydroxy-2-naphthoyl)-6-phenyl-2-thioxo-1,2-dihydropyridine-3-carbonitrile (**45c**)

It was obtained as yellow crystals from ethanol: M.P.: 136–138 °C, yield: 3.60 g (88%). IR (KBr) (*v*_max_/cm^−1^): 3390 (OH), 3255 (NH), 3059 (Ar–H), 2191 (CN), 1635 (C=O). MS (EI, 70 eV): *m*/*z* (%) = 494 (M^+^ + 2). ^1^H NMR (400 MHz, DMSO-d_6_): *δ* (ppm) 7.23–8.73 (m, 16H, aromatic H and NH), 8.74 (s, 1H, OH). Anal. calcd for C_29_H_17_ClN_2_O_2_S (492): C, 70.65; H, 3.48; N, 5.68. Found: C, 70.67; H, 3.50; N, 5.71.

### General procedure for preparation of compounds (49a,b)

A mixture of **3** (0.01 mol, 2.90 g) and 2-cyano-2-cyclopentylidene-ethaneethioamide **46a** (0.01 mol, 1.66 g) or 2-cyano-2-cyclohexylidene-ethaneethioamide **46b** (0.01 mol, 1.88 g) in ethanol (30 ml) containing a catalytic amount of piperidine was heated under reflux for 24 h. The reaction mixture was allowed to cool and poured into crushed ice then acidified with HCl. The separated solid was filtered off, washed with water and crystallized from the proper solvent to give **(49a,b)**.

#### 10-(1-hydroxy-2-naphthoyl)-9-phenyl-7-thioxo-8-azaspiro[4.5]dec-9-ene-6-carbonitrile (**49a**)

It was obtained as pale yellow crystals from ethanol: M.P.: 180–182 °C, yield: 3.25 g (74%). IR (KBr) (*v*_max_/cm^−1^): 3387 (OH), 3248 (NH), 3059 (Ar–H), 2935–2854 (Aliph-H), 2183 (CN), 1627 (C=O). MS (EI, 70 eV): *m*/*z* (%) = 440 (M^+^ + 2). ^1^H NMR (400 MHz, DMSO-d_6_): *δ* (ppm) 0.85–1.91 (m, 4H, 2CH_2_), 1.95 (s, 1H, CH-pyridine), 2.00–2.94 (m, 4H, 2CH_2_), 7.14–8.69 (m, 11H, aromatic H), 8.70 (s, 1H, OH)), 9.40 (s, 1H, NH). ^13^C NMR (100 MHz, DMSO-d_6_): *δ* (ppm) 27.8, 27.8, 38.3, 38.3, 39.0, 57.8, 116.2, 119.2, 122.3, 123.3, 123.9, 126.1, 126.4, 126.9, 127.3, 127.3, 127.5, 127.8, 128.3, 128.3, 128.8, 132.5, 135.2, 155.3, 166.7, 193.2, 198.9. Anal. calcd for C_27_H_22_N_2_O_2_S (438): C, 73.95; H, 5.06; N, 6.39%. Found: C, 73.97; H, 5.08; N, 6.41%.

#### 5-(1-hydroxy-2-naphthoyl)-4-phenyl-2-thioxo-3-azaspiro[5.5]undec-4-ene-1-carbonitrile (**49b**)

It was obtained as pale yellow crystals from dioxane: M.P.: 278–280 °C, yield: 3.95 g (87%). IR (KBr) (*v*_max_/cm^−1^): 3394 (OH), 3325 (NH), 3062 (Ar–H), 2931–2854 (Aliph-H), 2191 (CN), 1643 (C=O). MS (EI, 70 eV): *m*/*z* (%) = 454 (M^+^ + 2). ^1^H NMR (400 MHz, DMSO-d_6_): *δ* (ppm) 0.86–1.02 (m, 4H, 2CH_2_), 1.76 (s, 2H, CH_2_), 1.96 (s, 1H, CH-pyridine), 2.00–2.94 (m, 4H, 2CH_2_), 6.70–8.71 (m, 11H, aromatic H), 9.23 (s, 1H, OH), 9.87 (s, 1H, NH). Anal. calcd for C_28_H_24_N_2_O_2_S (452): C, 74.31; H, 5.35; N,6.19%. Found: C, 74.33; H, 5.37; N,6.21%.

### General procedure for preparation of compounds (51a–c)

A cold suspension of aryl diazonium salts **50a–c** (0.02 mol, 0.85, 0.86 and 0.77 g) (prepared from 0.02 mol of aromatic amines with the appropriate quantities of sodium nitrite and hydrochloric acid) was gradually added to a cold solution (0–5 °C) of **3** (0.002 mol, 1.45 g) in ethanol (50 ml) containing anhydrous sodium acetate (2 gm) with continuous stirring for 1 h. The resulting reaction product was filtered off, washed with water and crystallized from the proper solvent to give compounds **(51a–c)**.

#### 1-(1-hydroxynaphthalen-2-yl)-2-(2-(4-methoxyphenyl)hydrazono)-3-phenyl propane-1,3-dione (**51a**)

It was obtained as brown crystals from ethanol: M.P.: 170–172 °C, yield: 3.45 g (81%). IR (KBr) (*v*_max_/cm^−1^): 3420 (OH), 3400 (NH), 3062 (Ar–H), 2930 (Aliph-H), 1724, 1659 (2C=O). MS (EI, 70 eV): *m*/*z* (%) = 424 (M^+^). ^1^H NMR (400 MHz, DMSO-d_6_): *δ* (ppm) 3.87 (s, 3H, OCH_3_), 6.96–8.73 (m, 15H, aromatic H), 8.74 (s, 1H, OH), 11.00 (s, 1H, NH). Anal. calcd for C_26_H_20_N_2_O_4_ (424): C, 73.57; H, 4.75; N, 6.60. Found: C, 73.59; H, 4.77; N, 6.62.

#### 2-(2-(4-chlorophenyl)hydrazono)-1-(1-hydroxynaphthalen-2-yl)-3-phenylpropane-1,3-dione (**51b**)

It was obtained as brown crystals from ethanol: M.P.: 161–162 °C, yield: 3.30 g (77%). IR (KBr) (*v*_max_/cm^−1^): 3449 (OH), 3419 (NH), 3063 (Ar–H), 1723, 1650 (2C=O). MS (EI, 70 eV): *m*/*z* (%) = 430 (M^+^ + 2). ^1^H NMR (400 MHz, DMSO-d_6_): *δ* (ppm) 7.23–8.71 (m, 15H, aromatic H), 8.72(s, 1H, OH), 12.00 (s, 1H, NH). Anal. calcd for C_25_H_17_ClN_2_O_3_ (428): C, 70.01; H, 4.00; N, 6.53. Found:C, 70.02; H, 4.03; N, 6.55.

#### 1-(1-hydroxynaphthalen-2-yl)-3-phenyl-2-(2-p-tolylhydrazono) propane-1,3-dione (**51c**)

It was obtained as brown crystals from ethanol: M.P.: 166–168 °C, yield: 2.90 g (71%). IR (KBr) (*v*_max_/cm^−1^): 3422, 3400 (OH), 3063 (NH), 2926 (Aliph-H), 1723, 1650 (2C=O). MS (EI, 70 eV): *m*/*z* (%) = 408 (M^+^). ^1^H NMR (400 MHz, DMSO-d_6_): *δ* (ppm) 1.91 (s, 3H, CH_3_), 7.14–8.73 (m, 15H, aromatic H), 8.74 (s, 1H, OH)), 12.00 (s, 1H, NH). Anal. calcd for C_26_H_20_N_2_O_3_ (408): C, 76.45; H, 4.94; N, 6.86. Found: C, 76.46; H, 4.96; N, 6.88.

### General procedure for preparation of compounds (54a–c)

A mixture of compounds **51a–c** (0.001 mol, 0.424, 0.428 and 0.408 g), ammonium acetate (3 gm) and malononitrile (0.001 mol, 0.066 g) were fused for 10 min. The solid precipitate so formed was treated with ethanol and filtered out and crystallized from the proper solvent to give **(54a–c)**.

#### 6-(1-hydroxy-2-naphthoyl)-3-imino-2-(4-methoxyphenyl)-5-phenyl-2,3-dihydropyridazine-4-carbonitrile (**54a**)

It was obtained as brown crystals from dioxane: M.P.: 230–232 °C, yield: 3.75 g (79%). IR (KBr) (*v*_max_/cm^−1^): 3421 (OH), 3400 (NH), 3065 (Ar–H), 2928 (Aliph-H), 2200 (CN), 1650 (C=O). MS (EI, 70 eV): *m*/*z* (%) = 472 (M^+^). ^1^H NMR (400 MHz, DMSO-d_6_): *δ* (ppm) 3.99 (s, 3H, OCH_3_), 6.68–8.25 (m, 16H, aromatic H and NH), 8.71 (s, 1H, OH). Anal. calcd for C_29_H_20_N_4_O_3_ (472): C, 73.72; H, 4.27; N, 11.86. Found: C, 73.75; H, 4.29; N, 11.88.

#### 2-(4-chlorophenyl)-6-(1-hydroxy-2-naphthoyl)-3-imino-5-phenyl-2,3-dihydropyridazine-4-carbonitrile (**54b**)

It was obtained as brown crystals from dioxane: M.P.: 258–260 °C, yield: 3.60 g (75%). IR (KBr) (*v*_max_/cm^−1^): 3406 (OH), 3400 (NH), 3063 (Ar–H), 2200 (CN), 1650 (C=O). MS (EI, 70 eV): *m*/*z* (%) = 478 (M^+^ + 2). ^1^H NMR (400 MHz, DMSO-d_6_): *δ* (ppm) 6.67–8.25 (m, 16H, aromatic H and NH), 8.71 (s, 1H, OH). Anal. calcd for C_28_H_17_ClN_4_O_2_ (476): C, 70.52; H, 3.59; N, 11.75%. Found: C, 70.55; H, 3.63; N, 11.78%.

#### 6-(1-hydroxy-2-naphthoyl)-3-imino-5-phenyl-2-p-tolyl-2,3-dihydropyridazine-4-carbonitrile (**54c**)

It was obtained as brown crystals from dioxane: M.P.: 272–274 °C, yield: 3.85 g (84%). IR (KBr) (*v*_max_/cm^−1^): 3400, 3385 (OH), 3062 (NH), 2963 (Aliph-H), 2202 (CN), 1650 (CO). MS (EI, 70 eV): *m*/*z* (%) = 457 (M^+^ + 1). ^1^H NMR (400 MHz, DMSO-d_6_): *δ* (ppm) 1.76 (s, 3H, CH_3_), 6.67–8.27 (m, 16H, aromatic H and NH), 8.72 (s, 1H, OH). Anal. calcd for C_29_H_20_N_4_O_2_ (456): C, 76.30; H, 4.42; N, 12.27. Found: C, 76.33; H, 4.45; N, 12.30.
